# Interspecific associations of dominant tree populations in a virgin old-growth oak forest in the Qinling Mountains, China

**DOI:** 10.1186/s40529-016-0139-5

**Published:** 2016-09-01

**Authors:** Zongzheng Chai, Caili Sun, Dexiang Wang, Wenzhen Liu

**Affiliations:** 1 College of Forestry, Northwest A & F University, No.3 Taicheng Road, Yangling, 712100 Shaanxi People’s Republic of China; 2 State Key Laboratory of Soil Erosion and Dryland Farming on the Loess Plateau, Northwest A & F University, Yangling, 712100 Shaanxi People’s Republic of China; 3 Institution of Forestry, Xiaolongshan Forest Experiment Bureau, Tianshui, 741020 Gansu People’s Republic of China

**Keywords:** Interspecific competition, *Quercus aliena* var. *acutiserrata* Maxim., Development stage, Association index, Distribution pattern

## Abstract

**Background:**

Understanding interspecific associations in old-growth forests will help to reveal mechanisms of interspecific replacement in the process of forest development and provide a theoretical basis for vegetation restoration and reestablishment. In this study, we analyzed interspecific associations of eleven dominant tree populations of varying development stages in an old-growth oak forest stand in the Qinling Mountains, China. We examined overall interspecific associations (multiple species) and pairwise interspecific associations (two species).

**Results:**

Interspecific competition was intense during forest development and was the main factor driving succession. Community structure appears to become more stable over time which supports the harsh-benign hypothesis that interspecific competition is more common in stable sites.

**Conclusion:**

Old growth oak (*Quercus* spp.) forests are distributed widely around the world in part due to oak being a typical K-selected species. K-selected species produce fewer, high-quality offspring with higher survival rates, strong competitive ability, and longevity. The resulting distribution shifted from clumped to random, likely as a result of intense interspecific competition creating ecological niche differentiation.

## Background

Interspecific associations are the foundation for the formation and evolution of ecological communities (Haukisalmi and Henttonen 1998; Maihaiti and Zhang 2014). They result from species interactions, food chain co-actions, as well as similar responses and adaptations to environmental forces (Ofomata et al. 1999; Wang et al. 2010). Species-specific trait differences and unique ecological strategies affect population dynamics and the functioning of entire ecological communities (Wiegand et al. 2007). Measuring interspecific associations can aid in understanding interactions between species, ecological relationships between species, and population dynamics (Cole 1949; Cabaret and Hoste 1998; Ofomata et al. 1999).

Stable forest or climax vegetation communities are formed by the replacement and development of plant communities. Dramatic shifts in species abundance and composition take place during forest development (Liu et al. 2014). Analyzing development pathways of old-growth forests can provide valuable information on the main drivers of forest development in the absence of anthropogenic influence (Abrams and Copenheaver 1999; Petritan et al. 2014). Species competition and interactions drive the process of forest development. Species that compete with each other are those that occur in the same seral community and require the same habitat conditions (Parrish and Bazzaz 1982). Research on interspecific associations of tree species in old-growth forests will help to reveal mechanisms of interspecific replacement in the process of development and provide a theoretical basis for vegetation restoration and reestablishment (Maihaiti and Zhang 2014; Wang et al. 2010).

Mixed forests dominated by oak (*Quercus* spp.) and pure stands of oak are widely distributed globally. However, oak forests have poor natural regeneration (Cowell et al. 2010; Galbraith and Martin 2005; McCune and Cottam 1985; Nowacki and Abrams 2008; Shotola et al. 1992). Prior researches have suggested that significant compositional changes are occurring in oak-dominated forests and *Quercus* spp. are being replaced by mesophytic, relatively shade-tolerant species such as maple (*Acer* spp.) (Crow 1988; Dech et al. 2008; Gardiner and Hodges 1988; Tanouchi et al. 1994; Thadami and Ashton 1995; Watt 1919). This dominance shift has significant implications for biodiversity and ecosystems function, and has become an important focus of research and management (McEwan et al. 2011). Will shade-tolerant species ultimately replace oak species and become the dominant canopy species? If this is the case, then why are oak forests still widely distributed and dominant globally? Those questions can be addressed through examining interspecific associations.

Distribution patterns of trees can provide information on structural characteristics and forest dynamics related to the development stage of the forest (Akhavan et al. 2012; Hao et al. 2007). Abundance and composition changes take place during forest development and there are periods of relatively intense interspecific competition that limit coexistence (Mooney et al. 2008) and affect distribution patterns. A major focus of ecological research is to understand the outcomes of biological interactions and ecological process by analyzing spatial distribution patterns and associations (Li et al. 2014a).

In the present study, we analyzed the interspecific associations of eleven dominant tree populations during the development of a virgin old-growth oak broad-leaved mixed forest stand in the Qinling Mountains, China. Overall interspecific association (multiple species) and pairwise interspecific association (two species) were examined. We aim to answer the following questions: (1) are there significant changes in interspecific associations during the development of the forest stand? (2) can interspecific associations contribute to some evidence to explain the dominance of oak forests worldwide? (3) are there significant differences in species distribution during the development of the forest stand? (4) how are species distribution patterns and interspecific associations related?

## Methods

### Study area

The Qinling Mountains are located in a transitional region between the subtropical zone and warm temperate zone of central China. This region has high biodiversity and is ecological important (Yu et al. 2014; Zhao et al. 2014). The forests in the region have been harvested since the 1950s and much of the area is now covered by secondary growth that has low productivity and community stability (Chai and Wang 2016; Chai et al. 2016a; Li et al. 2004). Few old-growth forest stands exist in the Qinling Mountains.

The study took place in the western area of the Qinling mounatins on Xiaolong Mountain (104°22′–106°43′ E, 33°30′–34°49′ N). Altitude ranges between 1442 and 2489 masl. The mountain range (EW length 212.50 km, NS width 146.50 km) is a watershed of the upper reaches of the Yangtze and Yellow rivers (Zhao et al. 2008). This area is typical natural secondary forest with high biodiversity and abundant tree cover. The region experiences a mild and humid continental monsoon climate with a mean annual temperature of 9.5 °C. The annual sunshine hours are 2098 and the frost-free period is 185 days. The annual rainfall is 460–800 mm most of which falls from July to September (Chai et al. 2016b).

### Data collection

A field survey was conducted in the core zone of Baihua forest region from July to September, 2011 in the Xiaolongshan Mountains. Vegetation is a virgin old-growth (>100 years) oak broad-leaved mixed forest, and was representative of the remaining old-growth oak forest at mid-altitude in the Xiaolongshan Mountains. We established a permanent 140 × 70 m plot with an average elevation of 1723 m and a stand density of 887.70 trees hm^−2^. The plot was established away from roads and villages, where human disturbance is limited (Chai et al. 2016b).

**Fig. 1:**
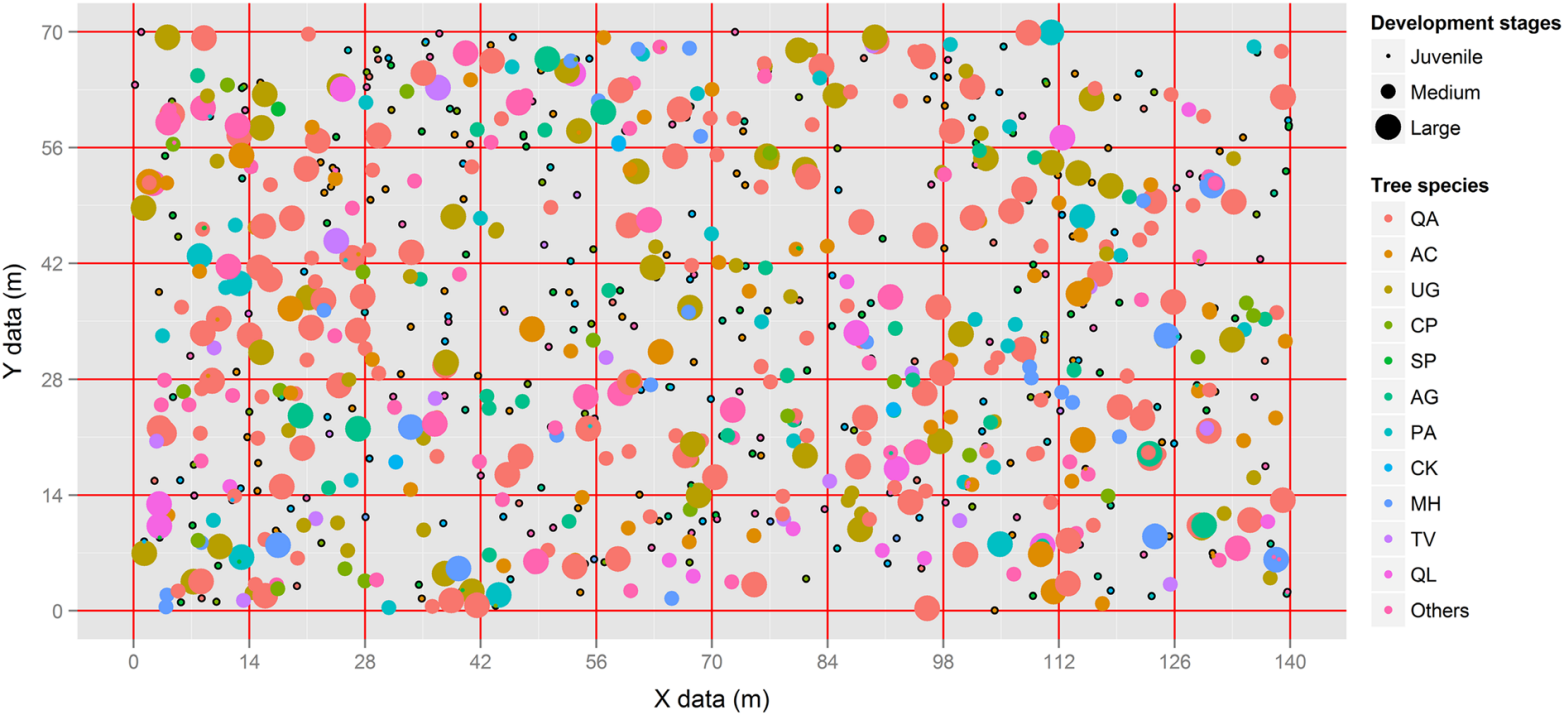
Spatial distributions of eleven dominant tree populations of different stages in a 0.98 ha (140 × 70 m) old-growth oak broad-leaved mixed forest plot in the Qinling Mountains, China. QA is *Quercus aliena* var. *acutiserrata* Maxim.; AC is *Acer caesium* subsp. *giraldii* (Pax) E. Murr.; UG is *Ulmus propinqua* Koidz.; CP is *Cerasus polytricha* (Koehne) Yü et Li; SP is *Symplocos paniculata* (Thunb.) Miq.; AG is *Acer ginnala* Maxim.; PA is *Pinus armandii* Franch.; CK is *Crataegus kansuensis* Wils.; MH is *Malus hupehensis* (Pamp.) Rehd.; TV is *Toxicodendron vernicifluum* (Stokes) F. A. Barkl.; QL is *Quercus wutaishanica* Blume. Juvenile tree means 5 cm ≤ DBH < 10 cm, Medium tree means 10 cm ≤ DBH < 25 cm, and large tree means DBH ≥ 25 cm

To accurately locate trees, the plot was divided into 50, 14 × 14 m subplots (Fig. 1). All trees with a diameter at breast height (DBH; 1.3 m) ≥5 cm were marked, and their locations were recorded using a total station (TOPCON-GTS-602AF, Fig. 1). Canopy cover, slope aspect, DBH, tree height, and species identity were recorded. To compare the structural differences among individuals within the same population, each tree was assigned a growth stage according to the size of the tree: juvenile (5 ≤ DBH < 10 cm), medium (10 ≤ DBH < 25 cm), and large (DBH ≥ 25 cm) (Chai et al. 2016b). This work was conducted based on Forestry Standards “Observation Methodology for Long-term Forest Ecosystem Research” of People’s Republic of China (LY/T 1952–2011).

### Data analysis

#### Importance values (IVs)

The Importance value (IV) of species is defined as the average of relative density (RD), relative frequency (RF), and relative dominance (Rd) of that species and was calculated using the following equations (Arbainsyah et al. 2014; Chai et al. 2016a, b):

$${\text{Density }}\left( {\text{D}} \right)\,{ =}\,\frac{\text{Number of individuals of a species}}{\text{Area of all sample units}}$$

$$\begin{aligned}{\text{Relative abundance }}\left( {RD} \right) = &\frac{\text{Number of individuals of a species}}{\text{Density for all species}}\\ &\times 100 \,{\% }\end{aligned}$$

$${\text{Frequency }}\left( F \right) = \frac{\text{Number of quadrats containing a certain specis}}{\text{Total number of quadrats}}$$

$${\text{Relative frequency }}\left( {RF} \right) = \frac{\text{Frequency of a certain species }}{\text{Total number of species}} \times 100\,{\% }$$

$${\text{Dominance }}(d) = \frac{\text{Basal area of a species}}{\text{Area of all sample units}}$$

$${\text{Relative dominance }}\left( {Rd} \right) = \frac{\text{Dominance of one specis}}{\text{Domiance of all species}} \times 100\,{\%}$$

$$IV = \left( {RD + RF + Rd} \right)/ 3$$

#### Contingency table

A 2 × 2 contingency table or species association table was generated (Table 1). For each pair of species A and B, we can obtain the following:

**Table 1:** 2 × 2 contingency table or species association table

		Species B		∑
		Present	Absent	
Species A	Present	a	b	m = a + b
	Absent	c	d	n = c + d
	∑	r = a + c	s = b + d	N = a + b + c + d

a: the number of samples in which species A and B co-occurred
b: the number of samples in which species A occurs, but not B
c: the number of samples in which species B occurs, but not A
d: the number of samples in which neither A nor B are found
N: the total number of samples.

#### Test of species association (two-species case)

To test for interspecific associations, a null hypothesis stating that species are independent was used. A corrected Chi square test (Yates’s correction formula) was used to test the null hypothesis of independence in the 2 × 2 contingency table (Yarranton 1966; Zaal 1993).

$$\chi^{2} = \frac{{N\left[ {\left| {ad - bc} \right| - {N \mathord{\left/ {\vphantom {N 2}} \right. \kern-0pt} 2}} \right]^{2} }}{{\left( {a + b} \right)\left( {c + d} \right)\left( {a + c} \right)\left( {b + d} \right)}}$$

When *χ* ^2^ < 3.841, there is no interspecific association; when 3.841 ≤ *χ* ^2^ < 6.635, there are certain associations between species; when *χ* ^2^ ≥ 6.635, there are significant associations between species. When *ad* > *bc*, the interspecific association is positive, and when *ad* < *bc*, the interspecific association is negative.

#### Measures of species association (two-species case)

To test the strength of associations and association coefficient (*AC*) index (Hurlbert 1969; Ofomata et al. 1999; Su et al. 2015) was used to further verify the results of the Chi square test.

When *ad* ≥ *bc* $AC = \frac{{\left( {ad - bc} \right)}}{{\left[ {\left( {a + b} \right)\left( {b + d} \right)} \right]}}$

When *ad* < *bc* and *d* ≥ *a* $AC = \frac{{\left( {ad - bc} \right)}}{{\left[ {\left( {a + b} \right)\left( {a + c} \right)} \right]}}$

When *ad* < *bc* and *d* < *a* $AC = \frac{{\left( {ad - bc} \right)}}{{\left[ {\left( {b + d} \right)\left( {c + d} \right)} \right]}}$

*AC* index assumes values from 1 for complete positive associations (*b* = 0, *c* = 0) to −1 for complete negative associations (*a* = 0, *d* = 0). It equals zero when there is no association.

#### Overall species association (multiple-species case)

The variance in total species number (or total density of individuals) in samples was compared to the sum of the variance of the individual species using the following equation (Schluter 1984):

$$V = \frac{{S_{T}^{2} }}{{\mathop \sum \nolimits \delta_{i}^{2} }}$$

$$S_{T}^{2}$$ is calculated:

$$s_{T}^{2} = \left( {{1 \mathord{\left/ {\vphantom {1 N}} \right. \kern-0pt} N}} \right)\sum\limits_{j}^{N} {\left( {T_{j} - t} \right)^{2} }$$

where *N* = the number of samples, *T*_*j*_ = the total number of species in sample *j*, and *t* = the mean number of species per sample.

$\delta_{i}^{2}$ is given by:

$$\delta_{i}^{2} = \left( {{1 \mathord{\left/ {\vphantom {1 N}} \right. \kern-0pt} N}} \right)\sum\limits_{j}^{N} {\left( {X_{ij} - t_{j} } \right)^{2} }$$

where *X*_*ij*_ = the abundance of species *i* in sample *j*; and *t*_*i*_ = the mean abundance of species *i*. When *V* = 1 under the assumption of independence, and *V* ≠ 1, indicates that the species tend to covary positively (*V* > 1) or negatively (*V* < 1) in their abundances. The significance of the association indices were assessed at *P* < 0.05 (Death 2000; Forbes et al. 1994; Schluter 1984).

#### Uniform angle index (*W*)

The uniform angle index (*W*) describes the degree of regularity for the four neighbors that are nearest to reference tree *i*. *W* is defined as the proportion of the angle (*α*) smaller than the standard angle *α*_0_ (72°), expressed as:

$$W_{i} = \frac{1}{4}\sum\limits_{j = 1}^{n} {z_{ij} } ,\,z_{ij} = \left\{ {\begin{array}{*{20}l} 1 &\quad {{\text{if}}\,\alpha - {\text{angle}}\,{\text{is}}\,{\text{smaller}}\,{\text{than}}\,\alpha_{0} } \\ {0,} &\quad {\text{otherwise}} \\ \end{array} } \right\}$$

$$\bar{W} = \frac{1}{{N_{sp} }}W_{i} = \frac{1}{{4N_{sp} }}\sum\limits_{i = 1}^{{N_{sp} }} {\sum\limits_{j = 1}^{4} {z_{ij} } }$$

where *N*_*sp*_ is the number of trees of species sp in the community.

*W* has a series of flexible values at five different levels (0.00, 0.25, 0.50, 0.75, and 1.00), and the average uniform angle index ($\bar{W}$) for the random case was defined by the bounds (0.475, 0.517). A $\bar{W}$-value of less than 0.475 corresponds to a regular distribution and values exceeding 0.517 correspond to a clumped distribution (Gadow and Hui 2002; Li et al. 2014b). To eliminate edge effects and improve the accuracy of the uniform angle index, we established a 5 m buffer zone around the plot. In the statistical analysis, only the trees in the reduced window (130 × 60 m) were used as reference trees, and the individual trees in the buffer zone were only considered as nearest neighbors of the trees in the reduced window (Li et al. 2014b). This edge correction can individually evaluate each tree to determine whether all *n* nearest neighbors are truly located within the plot.

R version 3.1.3 (R Core Team 2015) was used for all statistical analyses. The species association indices were conducted using the “spaa” package (Zhang and Ma 2014), the spatial association and uniform angle index were conducted using “spatstat” package (Baddeley and Turner 2005) and “forestSAS” package (Chai 2016)

## Results

### Species composition and importance value (IV) characteristics

A total of 48 tree species were identified belonging to 29 genera and 16 families (Table 2). The families with the greatest number of species were Rosaceae (*N* = 12, 25.00 %), Aceraceae (*N* = 9, 18.75 %), Betulaceae (*N* = 4, 8.33 %), Fagaceae (*N* = 3, 6.25 %), Tiliaceae (*N* = 3, 6.25 %), and Ulmaceae (*N* = 3, 6.25 %). The number of juvenile tree species was 40 (27 genera, 16 families), medium was 29 (20 genera, 14 families), and large was 18 (12 genera, 9 families).

**Table 2:** Composition and importance value (IV) index of the tree species in different development stages in an old-growth oak broad-leaved mixed forest

Species	Family	Density (trees ha^−1^)	Importance value (%)			
			Juvenile	Medium	Large	All
*Quercus aliena* var. *acutiserrata* Maxim	Fagaceae	190.82	4.79 ± 0.08	31.57 ± 0.18	50.7 ± 0.28	27.13 ± 0.1
*Acer caesium* subsp. *giraldii* (Pax) E.Murr	Aceraceae	137.76	25.5 ± 0.17	13.75 ± 0.14	4.59 ± 0.16	13.6 ± 0.08
*Ulmus propinqua* Koidz	Ulmaceae	77.55	0.72 ± 0.03	12.04 ± 0.15	20.79 ± 0.25	12.56 ± 0.09
*Cerasus polytricha* (Koehne) Yü et Li	Rosaceae	60.2	9.94 ± 0.11	6.26 ± 0.1	–	5.58 ± 0.05
*Symplocos paniculata* (Thunb.) Miq.	Symplocaceae	58.16	14.47 ± 0.13	0.54 ± 0.04	–	4.81 ± 0.04
*Acer ginnala* Maxim.	Aceraceae	57.14	6.66 ± 0.1	8.46 ± 0.12	1.93 ± 0.06	6.67 ± 0.05
*Pinus armandii* Franch.	Pinaceae	56.12	7 ± 0.11	7.07 ± 0.09	2.62 ± 0.07	6.18 ± 0.06
*Crataegus kansuensis* Wils	Rosaceae	47.96	14.73 ± 0.14	0.39 ± 0.02	–	4.7 ± 0.04
*Malus hupehensis* (Pamp.) Rehd	Rosaceae	32.65	1.21 ± 0.04	5.19 ± 0.1	3.93 ± 0.12	3.73 ± 0.05
*Toxicodendron vernicifluum* (Stokes) F. A. Barkl.	Anacardiaceae	22.45	0.89 ± 0.04	4.55 ± 0.08	0.56 ± 0.03	2.6 ± 0.04
*Quercus wutaishanica* Blume	Fagaceae	18.37	0.23 ± 0.02	2.52 ± 0.07	3.32 ± 0.1	2.95 ± 0.06
*Tilia paucicostata* Maxim	Tiliaceae	12.24	0.88 ± 0.03	0.77 ± 0.03	0.74 ± 0.03	0.98 ± 0.02
*Acer cappadocicum* Gled	Aceraceae	11.22	1.04 ± 0.04	1.13 ± 0.03	–	0.72 ± 0.02
*Lindera obtusiloba* Bl.	Lauraceae	11.22	1.66 ± 0.05	0.83 ± 0.03	–	0.87 ± 0.02
*Acer davidii* Franch.	Aceraceae	10.2	1.17 ± 0.04	1.08 ± 0.03	–	0.79 ± 0.02
*Morus alba* Linn.	Moraceae	10.2	1.48 ± 0.04	0.1 ± 0.01	–	0.62 ± 0.02
*Acer tetramerum* var. *betulifolium* (Maxim.) Rehd	Aceraceae	7.14	1.22 ± 0.04	–	–	0.36 ± 0.01
*Carya cathayensis* Sarg	Juglandaceae	6.12	0.77 ± 0.03	0.27 ± 0.02	1.53 ± 0.08	0.84 ± 0.03
*Kalopanax septemlobus* (Thunb.) Koidz	Araliaceae	5.1	0.55 ± 0.03	0.71 ± 0.03	–	0.41 ± 0.02
*Tilia oliveri* Szyszyl	Tiliaceae	4.08	–	0.44 ± 0.02	0.48 ± 0.02	0.4 ± 0.01
*Meliosma cuneifolia* var. *glabriuscula* Cufod	Sabiaceae	4.08	0.48 ± 0.02	–	–	0.16 ± 0.01
*Acer elegantulum* Fang et P. L. Chiu	Aceraceae	4.08	0.3 ± 0.02	0.29 ± 0.02	0.26 ± 0.02	0.27 ± 0.01
*Padus racemosa* (Linn.) Gilib	Rosaceae	3.06	0.43 ± 0.02	0.1 ± 0.01	–	0.14 ± 0.01
*Amygdalus persica* L.	Rosaceae	3.06	0.28 ± 0.02	0.34 ± 0.02	0.32 ± 0.02	0.34 ± 0.02
*Ulmus macrocarpa* Hance	Ulmaceae	3.06	–	0.29 ± 0.02	0.43 ± 0.03	0.31 ± 0.02
*Tilia oliveri*	Tiliaceae	2.04	0.31 ± 0.02	–	–	0.09 ± 0
*Betula platyphylla* Suk	Betulaceae	2.04	–	0.13 ± 0.01	0.41 ± 0.03	0.2 ± 0.01
*Corylus heterophylla* var. *sutchuenensis* Franch	Betulaceae	2.04	0.18 ± 0.01	–	–	0.08 ± 0.01
*Pyrus betulifolia* Bge.	Rosaceae	2.04	0.22 ± 0.02	–	0.37 ± 0.03	0.22 ± 0.01
*Acer henryi* Pax	Aceraceae	2.04	–	0.42 ± 0.02	–	0.17 ± 0.01
*Pyrus xerophila* Yü	Rosaceae	2.04	0.31 ± 0.02	–	–	0.09 ± 0
*Corylus heterophylla* Fisch	Betulaceae	2.04	0.48 ± 0.02	–	–	0.13 ± 0.01
*Spiraea alpina* Pall	Rosaceae	2.04	0.14 ± 0.01	0.19 ± 0.01	–	0.12 ± 0.01
*Rhus potaninii* Maxim.	Anacardiaceae	2.04	–	0.22 ± 0.02	0.32 ± 0.02	0.18 ± 0.01
*Pinus tabulaeformis* Carr.	Pinaceae	2.04	–	–	0.72 ± 0.04	0.33 ± 0.02
*Staphylea holocarpa* Hemsl.	Staphyleaceae	1.02	0.29 ± 0.02	–	–	0.06 ± 0
*Quercus spinosa* David ex Franchet	Fagaceae	1.02	0.21 ± 0.02	–	–	0.09 ± 0.01
*Celtis koraiensis* Nakai	Ulmaceae	1.02	0.16 ± 0.01	–	–	0.05 ± 0
*Sorbus hupehensis* Schneid.	Rosaceae	1.02	0.11 ± 0.01	–	–	0.04 ± 0
*Morus australis* Poir.	Moraceae	1.02	0.16 ± 0.01	–	–	0.04 ± 0
*Acer palmatum* Thunb.	Aceraceae	1.02	0.17 ± 0.01	–	–	0.04 ± 0
*Prunus salicina* Linn.	Rosaceae	1.02	0.18 ± 0.01	–	–	0.05 ± 0
*Carpinus cordata* Bl.	Betulaceae	1.02	0.15 ± 0.01	–	–	0.03 ± 0
*Fraxinus paxiana* Lingelsh.	Oleaceae	1.02	0.18 ± 0.01	–	–	0.06 ± 0
*Acer tsinglingense* Fang et Hsieh.	Aceraceae	1.02	–	0.12 ± 0.01	–	0.07 ± 0
*Sorbus alnifolia* (Sieb. et Zucc.) K. Koch	Rosaceae	1.02	0.2 ± 0.01	–	–	0.04 ± 0
*Amelanchier sinica* (Schneid.) Chun	Rosaceae	1.02	0.15 ± 0.01	–	–	0.04 ± 0
*Fraxinus platypoda* Oliver	Oleaceae	1.02	–	0.22 ± 0.02	–	0.06 ± 0

Juvenile tree means 5 cm ≤ DBH < 10 cm, Medium tree means 10 cm ≤ DBH < 25 cm, Large tree means DBH ≥ 25 cm, and All refers to all tree populations with DBH ≥ 5 cm in the studied plot

We identified *Quercus aliena* var. *acutiserrata* Maxim., *Acer caesium* subsp. *giraldii* (Pax) E.Murr., *Ulmus propinqua* Koidz., *Cerasus polytricha* (Koehne) Yü et Li, *Symplocos paniculata* (Thunb.) Miq., *Acer ginnala* Maxim., *Pinus armandii* Franch., *Crataegus kansuensis* Wils., *Malus hupehensis* (Pamp.) Rehd., *Toxicodendron vernicifluum* (Stokes) F. A. Barkl., and *Quercus wutaishanica* Blume as the dominant tree populations (first 11 tree populations in Table 2) accounting for 85.5 % of the total overstory density (DBH ≥ 5 cm).

Species importance values showed obvious differences at different development stages. The tree species *Q. aliena* var. *acutiserrata*, *U. propinqua*, and *Q. wutaishanica* increased, while, *A. caesium* subsp. *giraldii*, *P. armandii*, *T. paucicostata*, and *A. elegantulum* decreased as the forest stand developed (Table 2).

### Population structure of dominant tree populations

**Fig. 2:**
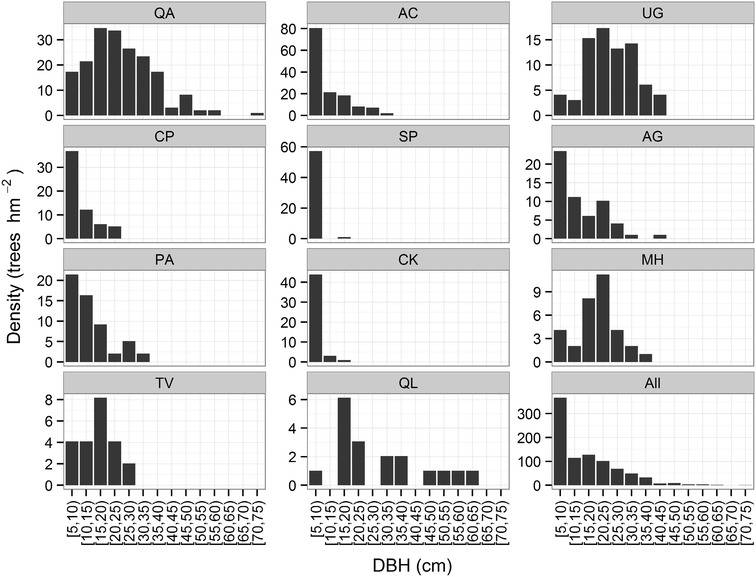
The DBH class distributions of eleven dominant tree populations and total forest (All) in an old-growth oak broad-leaved mixed forest in the Qinling Mountains, China. See above for abbreviations

The forest stand showed a reverse J-shaped distribution of tree diameters when all species were pooled. A total of 41.30 % of individuals were juvenile trees with a DBH class of 5–10 cm (Fig. 2). Extra-large (DBH ≥ 45 cm) trees species were concentrated in populations of *Q. aliena* var. *acutiserrata*, *U. propinqua*, *A. ginnala* and *Q. wutaishanica* (Fig. 2).

The 11 dominant tree populations showed great variability in abundance at different growth stages (Fig. 2). The size frequency distribution of the tree species indicated that *Q. aliena* var. *acutiserrata*, *U. propinqua*, *M. hupehensis*, *T. vernicifluum*, and *Q. wutaishanica* had a unimodal distribution. While, *A. caesium* subsp. *giraldii*, *C. polytricha*, *S. paniculata*, *A. ginnala*, *P. armandii*, and *C. kansuensis* showed an inverse J-shaped population structure suggesting they were the most dominant juvenile trees (Fig. 2).

### Overall species associations

The overall interspecific association of 11 dominant tree populations presented in Table 3. For the total dominant tree populations, the overall negative association (*V* < 1) was significant.

**Table 3:** The overall association among dominant tree populations in different development stages

Development stages	*δ*_*i*_^2^	*S*_*T*_^2^	Variance ratio (*V*)	W statistic	*χ* ^2^ ($\chi_{0.95}^{2}$, N, $\chi_{0.05}^{2}$, N)	Overall association
Juvenile	1.785	1.320	0.740	36.979	(34.765,67.505) N = 50	No significant negative
Medium	1.918	1.560	0.813	40.678		No significant negative
Large	1.003	0.680	0.678	31.857		Significantly negative
All	2.130	1.434	0.673	33.659		Significantly negative

For both the juvenile and medium tree stages the association was not significant. By contrast, large trees showed a significantly negative overall association. The overall association among different development stages was negative and suggests that the interspecific competition increases with stand development.

### Test of species association

**Fig. 3:**
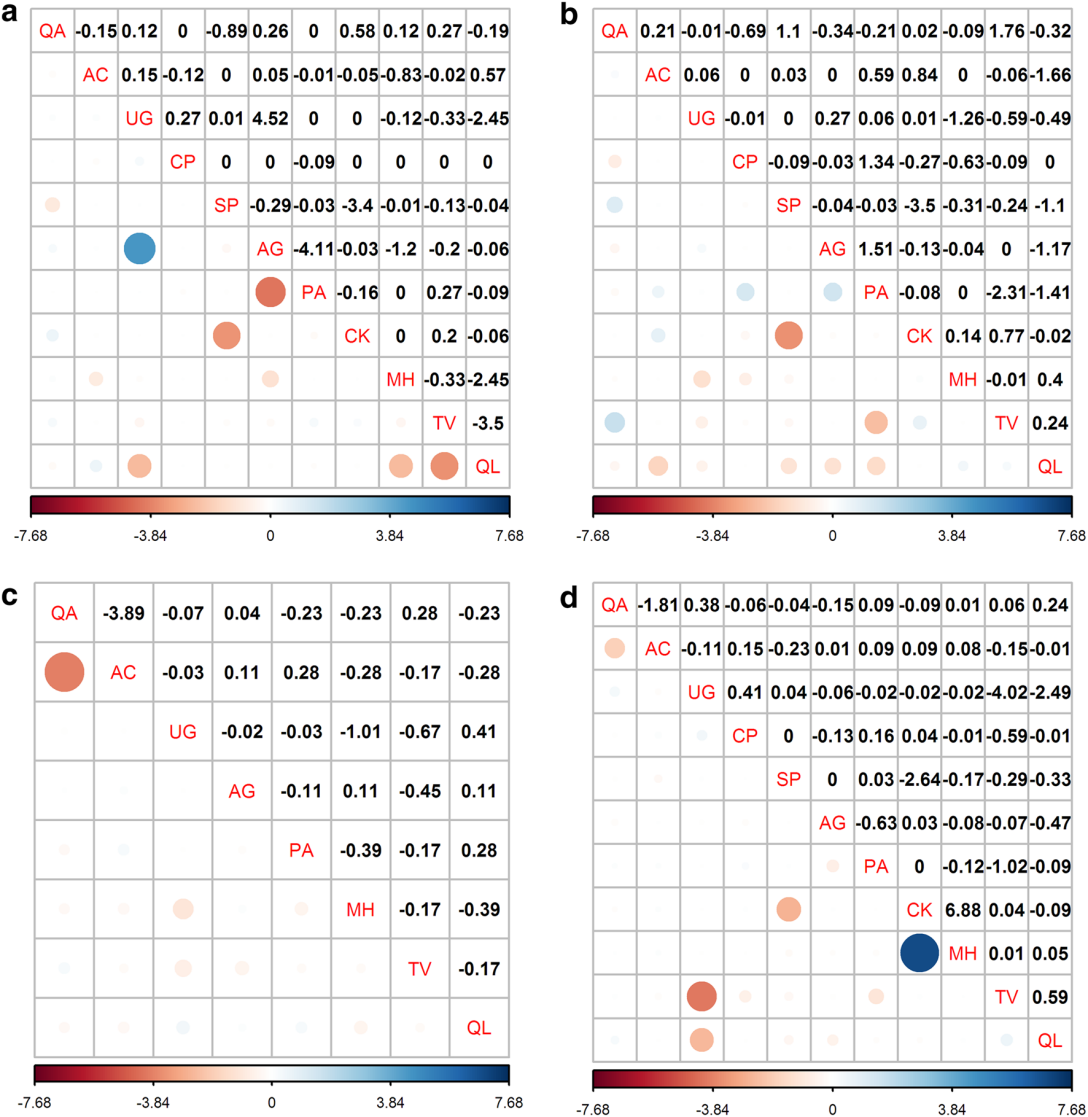
Semi-matrix graph of interspecific correction χ^2^ test of association of dominant tree populations in an old-growth oak broad-leaved mixed forest in the Qinling Mountains, China. **a** is development stage of juvenile tree (5 cm ≤ DBH < 10 cm); **b** is development stage of medium tree (10 cm ≤ DBH < 25 cm); **c** is development stage of large tree (DBH ≥ 25 cm); and **d** is total forest (DBH ≥ 5 cm). When χ^2^ ≥ 6.635, extra significant positive association; 3.841 ≤ χ^2^ < 6.635, significant positive association; −3.841 ≤ χ^2^ < 3.841, no association, independent distribution; −6.635 ≤ χ^2^ < −3.841, significant negative association; χ^2^ < −6.635, Extra significant negative association. See above for abbreviations

The *χ* ^2^ test showed that among total dominant tree populations, 21 pairs (38.18 %) showed a positive association, 31 pairs (56.36 %) showed a negative association, and 3 pairs (5.45 %) showed no association (Fig. 3d). A statistically significant positive association was found between *C. kansuensis* and *M. hupehensis* (*χ* ^2^ = 6.88, *P* < 0.01), and a significantly negative association between *S. paniculata* and *C. kansuensis* (*χ* ^2^ = −4.02, 0.01 < *P *< 0.05).

The proportion of negative associations increased slightly from juvenile (52.70 %) to medium (56.40 %) to large (71.40 %) trees suggesting that negative associations increase with stand age (Fig. 3a–c). In the juvenile tree populations, significantly positive associated pair were *U. propinqua* and *A. ginnala* (*χ* ^2^ = 4.52, 0.01 < *P* < 0.05), and negative associated pair were *A. ginnala* and *P. armandii* (*χ* ^2^ = −4.11, 0.01 < *P* < 0.05). In the medium tree populations there were no significantly associated pairs. In the large tree populations the significantly positive associated pair was *Q. aliena* var. *acutiserrata* and *A. caesium* subsp. *giraldii* (*χ* ^2^ = −3.89, 0.01 < *P* < 0.05). Overall, there were few significant associations among dominant tree populations. This suggests that species associations were weak for most species pairs, and the distribution of tree species is independent.

### Measures of species association

**Fig. 4:**
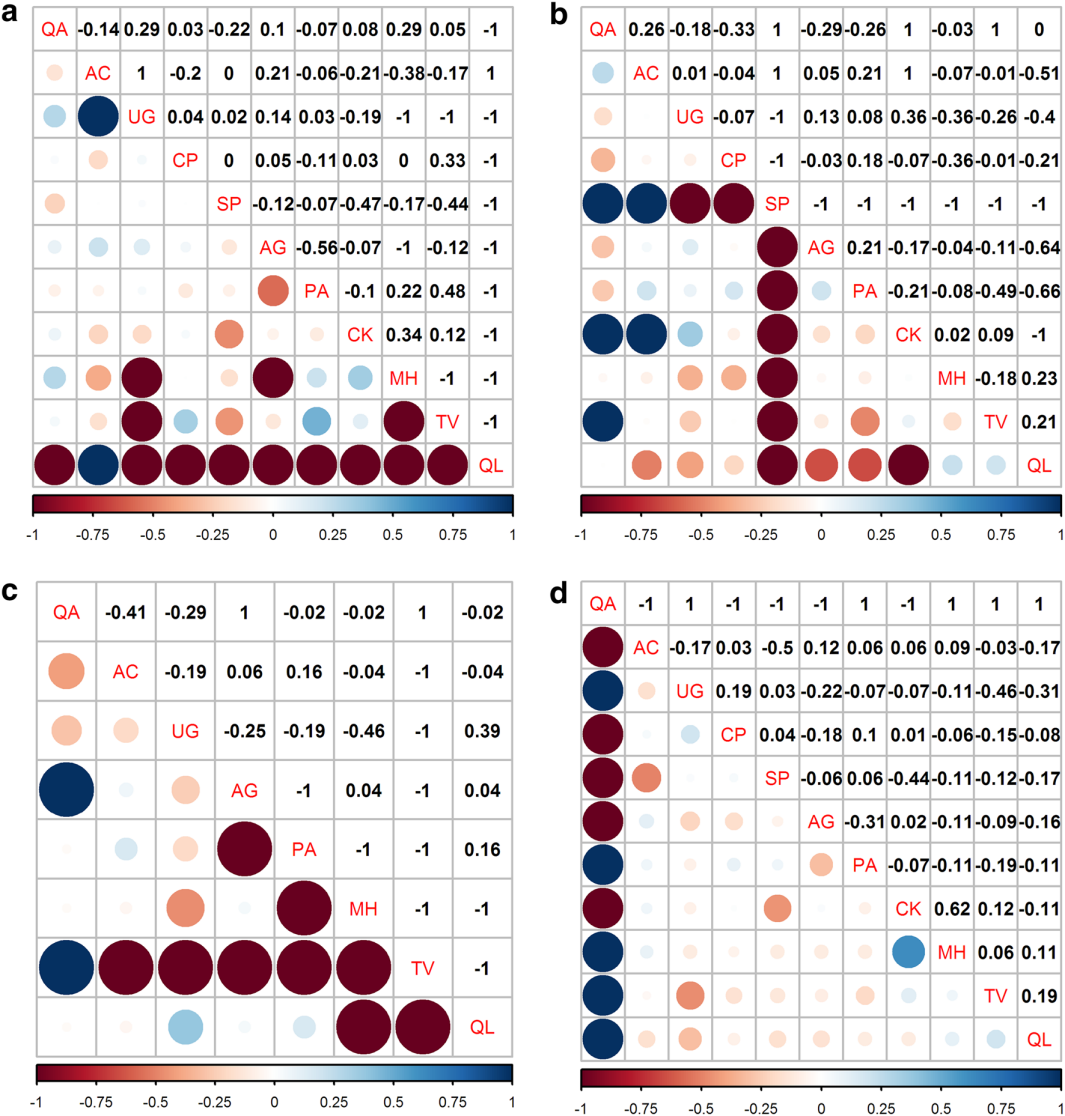
Semi-matrix graph of AC interspecific coefficient of dominant tree populations in an old-growth oak broad-leaved mixed forest in the Qinling Mountains, China. **a** is development stage of juvenile tree (5 cm ≤ DBH < 10 cm); **b** is development stage of medium tree (10 cm ≤ DBH < 25 cm); **c** is development stage of large tree (DBH ≥ 25 cm); and **d** is total forest (DBH ≥ 5 cm). See above for abbreviations

The *AC* index showed that among total dominant tree populations, 22 pairs (40 %) showed positive association and 33 pairs (60 %) showed negative associations. Highly positive associations (0.5 ≤ *AC* ≤ 1) were *Q. aliena* var. *acutiserrata* with *U. propinqua*, *P. armandii*, *M. hupehensis*, *T. vernicifluum*, and *Q. wutaishanica*; and the species pair *C. kansuensis* and *M. hupehensis*. Highly negative associations (−1 ≤ *AC* ≤ −0.5) were *Q. aliena* var. *acutiserrata* with *A. caesium* subsp. *giraldii*, *C. polytricha*, *S. paniculata*, *A. ginnala*, and *C. kansuensis*. The *AC* index showed that species pair *C. kansuensis* and *M. hupehensis* had the higher positive association (*AC* = 0.62), and *U. propinqua* and *T. vernicifluum* had the higher negative association (*AC* = −0.46), which was consistent with the results of the *χ* ^2^ test (Fig. 4d).

The *AC* index of juvenile trees showed that 20 pairs (36.36 %) had positive association, 32 pairs (58.18 %) showed negative association, and 3 pairs (5.45 %) had no association. Highly positive associations (0.5 ≤ *AC* ≤ 1) of *A. caesium* subsp. *giraldii* were found with *U. propinqua* and *Q. wutaishanica*. Highly negative associations (−1 ≤ *AC* ≤ −0.5) of *Q. wutaishanica* were found with most other species except *A. caesium* subsp. *giraldii*, and the species pairs *U. propinqua* and *M. hupehensis*, *U. propinqua* and *T. vernicifluum*, *A. ginnala* and *P. armandii*, *A. ginnala* and *M. hupehensis*, and *M. hupehensis* and *T. vernicifluum* also exhibited highly negative associations. *AC* index showed that the species pair *U. propinqua* and *A. ginnala* had positive association (*AC* = 0.14), and *U. propinqua* and *T. vernicifluum* had negative association (*AC* = −0.56), which were consistent with the results of the *χ* ^2^ test (Fig. 4a).

In the development stage of medium trees, *AC* association showed that 18 pairs (32.73 %) showed positive association, 36 pairs (65.46 %) showed negative association, and 1 (1.82 %) was no association. Highly positive associations (0.5 ≤ *AC* ≤ 1) were *Q. aliena* var. *acutiserrata* with *S. paniculata*, *C. kansuensis*, and *T. vernicifluum*; *A. caesium* subsp. *giraldii* with *S. paniculata*, *C. kansuensis*. Highly negative associations (−1 ≤ *AC* ≤ −0.5) were *Q. wutaishanica* with *A. caesium* subsp. *giraldii, S. paniculata*, *A. ginnala*, *P. armandii* and *C. kansuensis*; *S. paniculata* with *Q. aliena* var. *acutiserrata*, *C. polytricha*, *A. ginnala*, *P. armandii*, *C. kansuensis*, *M. hupehensis*, *T. vernicifluum*, (Fig. 4b).

In the development stage of large trees, *AC* association showed that 8 pairs (28.57 %) showed positive association, 20 pairs (71.43 %) showed negative association. Highly positive associations (0.5 ≤ *AC* ≤ 1) were *Q. aliena* var. *acutiserrata* with *A. ginnala*, *T. vernicifluum*. Highly negative association (−1 ≤ *AC* ≤ −0.5) were *T. vernicifluum* with *A. caesium* subsp. *giraldii*, *U. propinqua*, *A. ginnala*, *P. armandii*, *M. hupehensis*, and *Q. wutaishanica*; *M. hupehensis* with *Q. wutaishanica*, *P. armandii*; and the species pair *A. ginnala* and *P. armandii*. *AC* index showed that *Q. aliena* var. *acutiserrata* and *A. caesium* subsp. *giraldii* with higher negative association (*AC* = −0.41), which was consistent with the results of the *χ* ^2^ test (Fig. 4c).

## Discussion

**Fig. 5:**
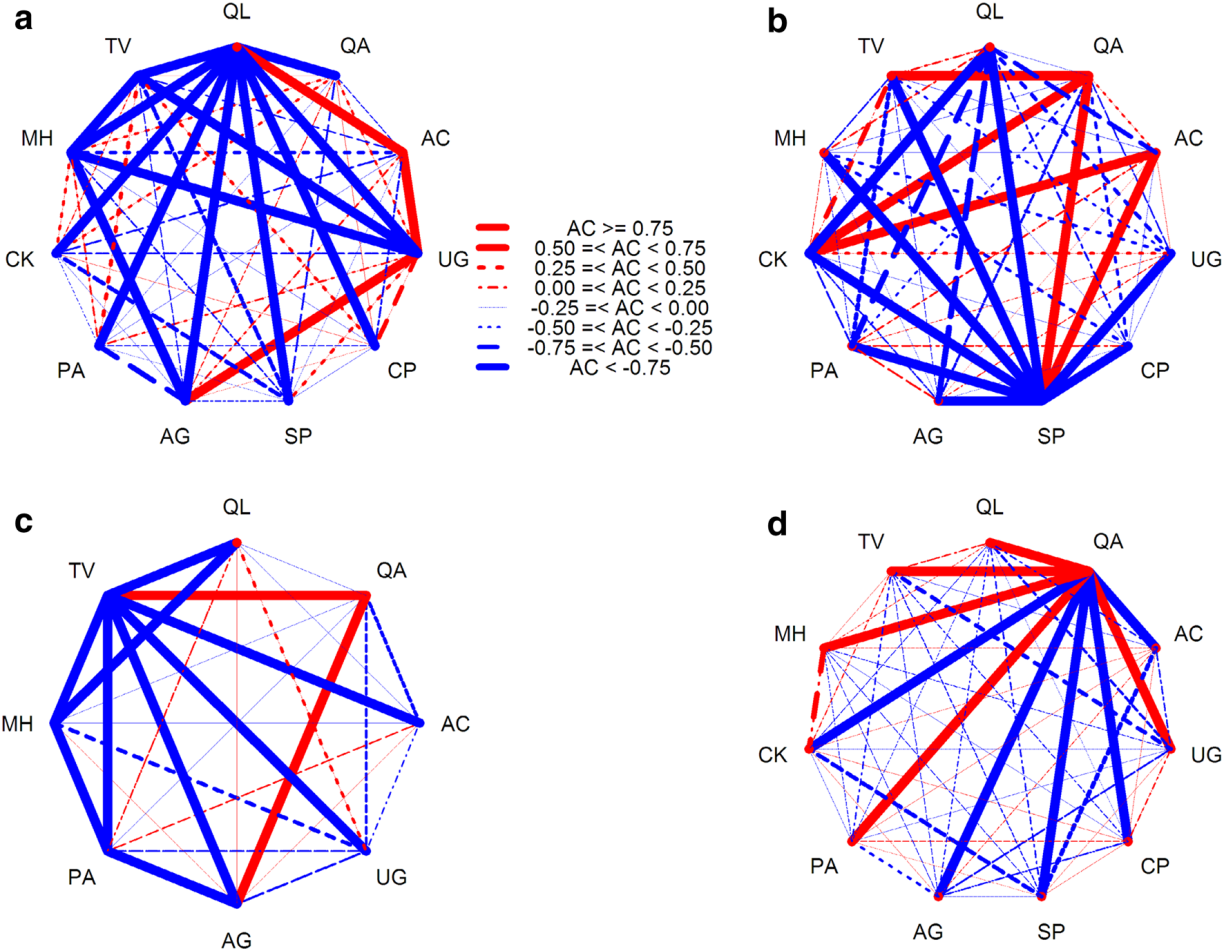
Network of Association coefficient index in an old-growth oak broad-leaved mixed forest in the Qinling Mountains, China. **a** is development stage of juvenile tree (5 cm ≤ DBH < 10 cm); **b** is development stage of medium tree (10 cm ≤ DBH < 25 cm); **c** is development stage of large tree (DBH ≥ 25 cm); and **d** is total forest (DBH ≥ 5 cm). See above for abbreviations

Interspecific competition increased with the development of the old-growth oak broad-leaved mixed forest stand. Negative associations among species are indicative of interspecific competition (Rejmánek and Lepš 1996). Negative interspecific associations dominated overall interspecific association (multiple species) and pairwise interspecific association (two species) in the old-growth oak broad-leaved mixed forest of the Xiaolong Mountains. This suggests that interspecific competition is very intense, which is similar to the findings by Ma et al. (2014) and Fraver et al. (2014) who found that interspecific competition continues to influence forest processes and structure in undisturbed old-growth forests. The increasing negative associations with increasing forest stages are consistent with the harsh-benign hypothesis (Peckarsky 1983). The harsh-benign hypothesis predicts that competitive interactions are more likely in stable environments and negative interspecific associations occur more often in stable sites than the unstable sites (Death 2000; Peckarsky 1983). The same results have been reported in many other studies (Hao et al. 2007; Liu et al. 2014). Old-growth oak broad-leaved mixed forests over 100 years old have reached a late-successional stage and the *AC* index network corroborated this theory (Fig. 5). Forests dominated by *Q. aliena* var. *acutiserrata*, *T. vernicifluum*, and *A. ginnala,* are typical stable forest communities distributed in mid-mountain zones of Qinling Mountains (Lei et al. 1996). Our results further support the harsh-benign hypothesis that species interactions are more common in stable sites.

Oak forests are distributed widely around the world, in part because it is a typical K-selected species. Mixed and pure stands of oak can adapt to a wide variety of site and soil conditions (Abrams et al. 1998; Alvarez et al. 2009; Collins and Battaglia 2008), however, globally *Quercus* species generally have poor natural regeneration in the forests where they occur (Crow 1988; Dech et al. 2008; Gardiner and Hodges 1988; Tanouchi et al. 1994; Thadami and Ashton 1995; Watt 1919) and this same trend was observed in the oak forests of the Qinling Mountains (Chai and Wang 2016; Yu et al. 2013a). Yu et al. (2013b) noted that there could be strong competition for seed dispersers among co-occurring species, so variation in seed size and other seed traits may shape the behavior of dispersers. *Quercus* are large seed species with a low seedset and is a rodent-dispersed species. Previous studies have shown that oak seeds were usually transported to adjacent pine forests by rodents, resulting poor natural regeneration in the oak forests where they occur (Chang et al. 2012; Yu et al. 2013b). The calculated importance values may support this phenomenon as there were significantly fewer juvenile oak trees than other species (Table 2). However, the hard shell and high nutritional content (e.g., protein, fat, and starch) of oak seeds (Chang et al. 2012) result in high-quality offspring and competitive ability for those that do establish. This phenomenon is reflected in the importance values of medium and large trees and results in its dominance in older forest stands (Table 2). In addition, long-living tree species are able to maintain their dominance in a forest stand even if they only regenerate successfully once over many years (Warner and Chesson 1985). The findings of Hou et al. (2004) in a *Quercus*-*Betula* forest in northern China showed that the longevity of *Quercus* combined with its dominance in the stand can compensate for the low regeneration and allow them to persist. Yu et al. (2013a) further corroborated this result by finding that *Q. aliena* var. *acutiserrata* regeneration was not affected by low establishment. These results all suggest that despite low regeneration, oak populations will maintain their dominance if there are no large-scale disturbances in the Qinling Mountains.

**Fig. 6:**
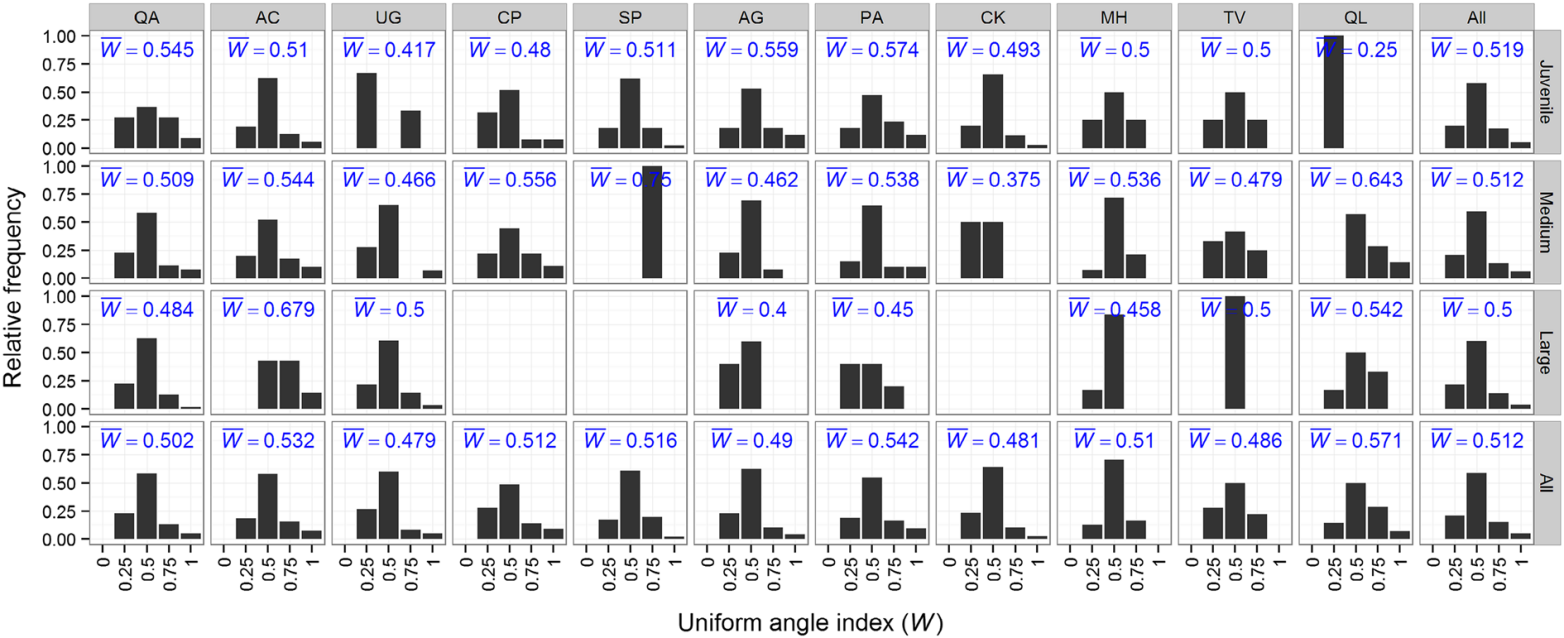
Distribution of uniform angle index for dominant tree populations in an old-growth oak broad-leaved mixed forest in the Qinling Mountains, China. $\bar{W}$ is the average value of uniform angle index. See above for abbreviations

Observed distribution patterns of tree species maybe the result of ecological niche differentiation due to the intense interspecific competition. Species interactions influence ecological processes such as growth, regeneration and mortality which in turn influence tree distribution (Bieng et al. 2013; Kang et al. 2014). Our results showed that there are obvious differences among the eleven dominant tree populations during the development of the forest stand but the overall distribution is random (Fig. 6). This suggests that the forest is in a stable state. Some researches explain this phenomenon as a long-term species interaction between the plant community and environment (Hao et al. 2007; Liu et al. 2014; Nathan 2006; Schoolmaster 2013). We conclude that the shift from clumped to random as the forest stand developed maybe evidence for niche differentiation and selection of species composition through the sieve of interspecific relations. This is supported by Zaal (1993) and Getzin et al. (2006) who found that spatial distribution and tree size are not independent patterns but are commonly affected by the interspecific competition. Parrish and Bazzaz (1982) showed that early successional species of plants have broad, overlapping niche occupation on many gradients, whereas later successional species show more niche differentiation. Call and Nilsen (2003) and Su et al. (2015) also support the theory that species pairs with positive associations share similar resources and exhibit a wide niche overlap, while negative association indicate that plants have different habitat and resource requirements.

## Conclusion

Old growth oak (*Quercus* spp.) forests are distributed widely around the world in part due to oak being a typical K-selected species. K-selected species produce fewer, high-quality offspring with higher survival rates, strong competitive ability, and longevity. Interspecific competition was intense during forest development and was the main factor driving succession, which supports the harsh-benign hypothesis that interspecific competition is more common in stable sites. The resulting distribution shifted from clumped to random, likely as a result of intense interspecific competition creating ecological niche differentiation.
